# Impact of Dabigatran versus Phenprocoumon on ADP Induced Platelet Aggregation in Patients with Atrial Fibrillation with or without Concomitant Clopidogrel Therapy (the Dabi-ADP-1 and Dabi-ADP-2 Trials)

**DOI:** 10.1155/2015/798486

**Published:** 2015-07-01

**Authors:** Amadea M. Martischnig, Julinda Mehilli, Janina Pollak, Tobias Petzold, Anette K. Fiedler, Katharina Mayer, Stefanie Schulz-Schüpke, Dirk Sibbing, Steffen Massberg, Adnan Kastrati, Nikolaus Sarafoff

**Affiliations:** ^1^Deutsches Herzzentrum München, Technische Universität München, 80636 Munich, Germany; ^2^Ludwig-Maximilians-Universität München, 80337 Munich, Germany; ^3^German Centre for Cardiovascular Research (DZHK), Partner Site Munich Heart Alliance, 80802 Munich, Germany

## Abstract

*Background.* A relevant number of patients receive triple therapy with clopidogrel, aspirin, and oral anticoagulation. Clopidogrel's efficacy on ADP induced platelet function may be influenced by concomitant antithrombotic therapies. Data regarding the effect of dabigatran on platelet function is limited to* in vitro* studies and healthy individuals.* Methods.* The “Dabi-ADP-1” and “Dabi-ADP-2” trials randomized patients with atrial fibrillation to either dabigatran or phenprocoumon for a 2-week period. In Dabi-ADP-1 (*n* = 70) patients with clopidogrel therapy were excluded and in Dabi-ADP-2 (*n* = 46) patients had to be treated concomitantly with clopidogrel. The primary endpoint was ADP-induced platelet aggregation between dabigatran and phenprocoumon at 14 days. Secondary endpoints were ADPtest HS-, TRAP-, and COL-induced platelet aggregation.* Results.* There was no significant difference regarding the primary endpoint between both groups in either trial (Dabi-ADP-1: Dabigatran: 846 [650–983] AU × min versus phenprocoumon: 839 [666–1039] AU × min, *P* = 0.90 and Dabi-ADP-2: 326 [268–462] versus 350 [214–535], *P* = 0.70) or regarding the secondary endpoints, ADPtest HS-, TRAP-, and COL-induced platelet aggregation.* Conclusion.* Dabigatran as compared to phenprocoumon has no impact on ADP-induced platelet aggregation in atrial fibrillation patients neither with nor without concomitant clopidogrel therapy.

## 1. Introduction

Dabigatran is at least as effective as vitamin K antagonists in the prevention of stroke and systemic embolism in patients with atrial fibrillation [1]. Dabigatran etexilate (Pradaxa) is an oral prodrug that is converted by serum esterases to dabigatran, a potent, direct, competitive inhibitor of thrombin. Thrombin has multiple roles in hemostasis. It converts fibrinogen to fibrin which is necessary to form the fibrous matrix of blood clots and it also has a direct action on cells [2]. Thrombin has an impact on shape and vascular permeability of vascular endothelium and is the most potent agonist for platelet activation and aggregation [2]. By inhibiting thrombin, platelet signaling pathways are also blocked and therefore platelet function may be affected.

Data regarding the effect of dabigatran on platelet function is limited to* in vitro* studies [3–5] and studies in healthy individuals of modest size [6] and has so far not been tested in real life patients.

Triple therapy, the combination of aspirin, clopidogrel, and oral anticoagulation, is necessary in patients with coronary stent implantation who also have atrial fibrillation to reduce ischemic events [7]. With the advent of the direct oral anticoagulants (DOAC), substances such as dabigatran etexilate are also given concomitantly with clopidogrel [8]. Platelet function testing is more and more emerging in the clinical routine because it has been shown that in patients who are treated with dual antiplatelet therapy (DAT) with aspirin and clopidogrel, both low and high on-treatment platelet reactivity (HPR) are associated with adverse clinical events [9]. One factor that may predispose to HPR is the patients' comedication that may interfere with clopidogrel metabolization. In fact, there is some evidence that traditional anticoagulants such as the vitamin K antagonist phenprocoumon attenuate the antiplatelet effects of clopidogrel [10].

In the setting of concomitant clopidogrel therapy, it was already demonstrated that the parenteral direct thrombin inhibitor bivalirudin was able to further inhibit ADP induced platelet aggregation [11, 12]. Whether this is due to a direct interaction with the platelets or has an impact on clopidogrel metabolization is unclear. The oral direct thrombin inhibitor dabigatran is currently challenging the role of vitamin K antagonists in patients with atrial fibrillation and in those treated with triple therapy [8]. Therefore evaluating its role on platelet function and clopidogrel metabolization in real life patients is imperative. It is therefore our aim to evaluate whether dabigatran as compared to phenprocoumon alters ADP mediated platelet signaling pathway in clopidogrel naive patients or in patients concomitantly treated with clopidogrel. We therefore initiated two randomized trials to study the impact of dabigatran as compared to phenprocoumon (i) on ADP induced platelet aggregation in patients with atrial fibrillation (Dabi-ADP-1) and (ii) on clopidogrel mediated ADP induced platelet aggregation in patients with atrial fibrillation who are concomitantly treated with clopidogrel (Dabi-ADP-2).

## 2. Methods

### 2.1. Study Population

The “Impact of DABIgatran and phenprocoumon on the ADP induced platelet aggregation in patients with atrial fibrillation” (DABI ADP 1) study and the “Impact of DABIgatran and phenprocoumon on the clopidogrel mediated ADP induced platelet aggregation in patients with atrial fibrillation” (Dabi-ADP-2) study were two single centre randomized open label trials designed to compare the impact of dabigatran etexilate (Pradaxa) versus the vitamin K antagonist phenprocoumon on platelet function (ClinicalTrials.gov identifiers: NCT01339819 and NCT01352702). Patients were enrolled at the Deutsches Herzzentrum Munich between April 2011 and February 2013.

Both trials shared the same inclusion and exclusion criteria with the main difference that in Dabi-ADP-1 patients with clopidogrel therapy were excluded and in Dabi-ADP-2 had to be treated concomitantly with clopidogrel.

Patients were eligible if they were 18 years of age, if they had atrial fibrillation with an indication for oral anticoagulation, and if written informed consent by the patient or her/his legally authorized representative for participation in the study was obtained. Exclusion criteria included patients with a recent thromboembolic event (defined as severe disabling stroke in the last 6 months or any stroke in the last 14 days) or a high thromboembolic risk such as a history of mechanical valve, pulmonary embolism, deep vein thrombosis, or LV thrombus. In addition, patients with a contraindication for oral anticoagulation, active bleeding or high bleeding risk, cardiogenic shock, severe renal insufficiency (Creatinine Clearance < 30 mL/min), or moderate or severe hepatic impairment (Child-Pugh class B or C) were excluded. The studies were conducted in accordance with the provisions of the Declaration of Helsinki and with the International Conference on Harmonization Good Clinical Practices. The trial protocols were approved by the institutional ethics committee responsible for the Deutsches Herzzentrum Munich (Germany) and the Bundesinstitut für Arzneimittel und Medizinprodukte (BfArM, Germany).

### 2.2. Study Protocol

Patients who met all of the inclusion criteria and none of the exclusion criteria were randomized in the order that they qualify. Allocation to treatment was made by means of sealed envelopes containing a computer-generated sequence. Patients were randomized according to a 1 × 1 factorial design to a dabigatran versus a phenprocoumon therapy for 2 weeks. Time zero was defined as the time of randomization. Patients were considered enrolled in the study and eligible for the final intention to treat analysis at the time of randomization. Commercially available drugs were given according to the protocol.

Oral anticoagulation with phenprocoumon or dabigatran was given on the day of randomization and was administered orally at the dosage recommended by current guidelines [13, 14]. Phenprocoumon was given with a target INR of 2.0–3.0 and dabigatran was administered at a dosage of 110 mg or 150 mg twice daily. Study drugs were provided by the sponsor (Deutsches Herzzentrum Munich). Other cardiac medications were given according to the judgment of the patients' physician (e.g., ASA, ß-blockers, ACE-inhibitors, statins, proton pump inhibitors, etc.).

Patients were scheduled for an outpatient visit at 14 days after randomization for clinical follow-up and laboratory testing (see below).

### 2.3. Laboratory Testing

Whole blood for platelet function testing on the Multiplate analyzer (Roche Diagnostics, Basel, Switzerland) was obtained from the cubical vein. Blood was placed in 4.5 mL plastic tubes containing the anticoagulant lepirudin (25 *μ*g/mL; Refludan, Dynabyte, Munich, Germany). Platelet aggregation was assessed with multiple electrode aggregation (MEA) using an impedance aggregometer (Multiplate analyzer). ADP (6.4 *μ*mol/L), ADPtest HS (6.4 *μ*mol/L ADP in the presence of 9.4 nmol/L prostaglandin E1), TRAP-6 (32 *μ*mol/L), and Collagen (3.2 *μ*g/mL collagen (COLtest)) served as agonists. Details of this method have been reported previously [15, 16]. Aggregation measured on the Multiplate device is quantified as area under the curve of aggregation units (AU) (area under the curve = AU × min). All material used for platelet function testing was obtained from the manufacturer.

### 2.4. Study Endpoints and Definitions

The primary endpoint of both studies was the ADP induced platelet aggregation in patients treated with dabigatran versus phenprocoumon treatment at 14 days. ADP was chosen as primary endpoint because there is a multitude of studies that have linked alterations in the ADP value associated with antithrombotic therapy with clinical events [9].

The secondary endpoints were ADPtest HS, TRAP, and COL induced platelet aggregation in patients treated with dabigatran versus patients with phenprocoumon treatment at 14 days.

Patients were further monitored throughout the two-week study period for the occurrence of adverse events such as death, stroke, myocardial infarction (MI) according to TIMI criteria [17], stent thrombosis, TIMI major, or TIMI minor bleeding [17]. The diagnosis of ischemic or haemorrhagic stroke required confirmation by computed tomography or magnetic resonance imaging of the head. Adjudication of bleeding events according to BARC criteria [18] was done in a retrospective manner.

### 2.5. Follow-Up

Detailed information regarding the occurrence of adverse events was obtained in this population during follow-up at 14 days in the outpatient clinic. Patients who could not come to the hospital were interviewed by phone. Those with cardiac complaints underwent a complete clinical, electrocardiographic, and laboratory check-up. General practitioners, referring cardiologists, patients, or their relatives were contacted for additional information if necessary.

Relevant data were collected from source documents and prospectively entered into a computerized database by specialized personnel of the data coordinating Intracoronary Stenting and Antithrombotic Research (ISAR) center.

### 2.6. Statistical Methods

There is some evidence that the parenteral direct thrombin inhibitor bivalirudin results in further inhibition of ADP induced platelet aggregation [12] while phenprocoumon attenuates clopidogrel mediated ADP aggregation [10]. Sample size calculation was therefore based on the assumption that administration of dabigatran as compared to phenprocoumon results in a 25% absolute decrease of maximal ADP. Choosing a power of 80% and a two-sided *α* value of 0.05 a sample size of at least 29 per group was required. To compensate for losses to follow-up, each study was designed to enroll a total of 70 patients (35 per group).

In Dabi-ADP-2 a blinded interim review performed in February 2013 demonstrated that more than 1000 patients would need to be included to show a significant difference between groups. On the basis of these data, the steering committee decided to terminate Dabi-ADP-2 prematurely for futility.

A comparison of categorical variables, expressed as counts (percentages), was performed using the Fisher exact or the *χ* ^2^ test, as appropriate. Continuous variables were expressed as means (±SD) and compared with the unpaired, 2-sided Student's *t*-test if normally distributed; otherwise, they were expressed as medians (25th–75th percentile) and statistically analysed by the Wilcoxon rank-sum test.

A *P* value <0.05 was considered to indicate statistical significance. All statistical analyses were performed with the software R (version 2.15.0; The R Foundation for Statistical Computing).

## 3. Results

### 3.1. Populations

**Figure 1:**
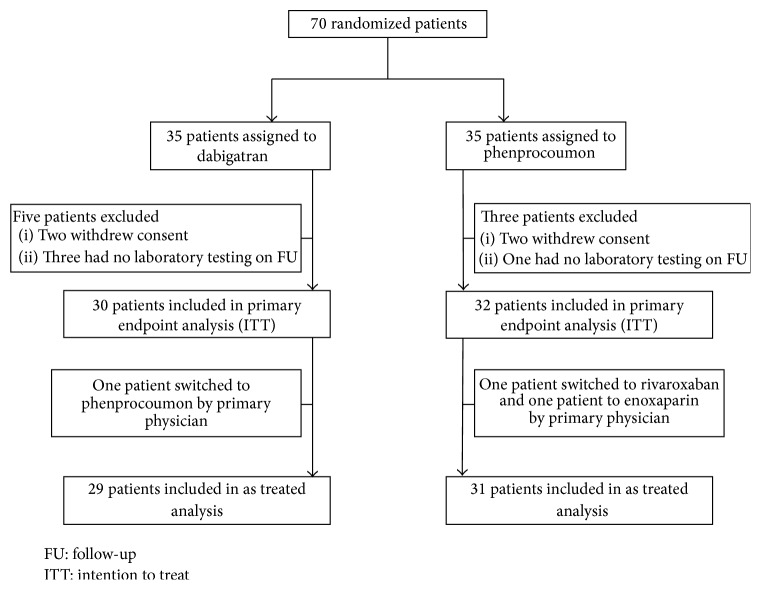
Patient population Dabi-ADP-1.

In Dabi-ADP-1, 70 patients were enrolled and randomized to receive dabigatran (*n* = 35) or phenprocoumon (*n* = 35). There was no significant difference in terms of baseline patient characteristics (Table 1). Median INR levels (interquartile range) at baseline were 1.1 (1.0-1.1) in the dabigatran group and 1.1 (1.1-1.2) in the phenprocoumon group. According to intention to treat analysis the primary and secondary endpoints could be analyzed in 30 patients in the dabigatran group and 32 patients in the phenprocoumon group (Figure 1).

**Figure 2:**
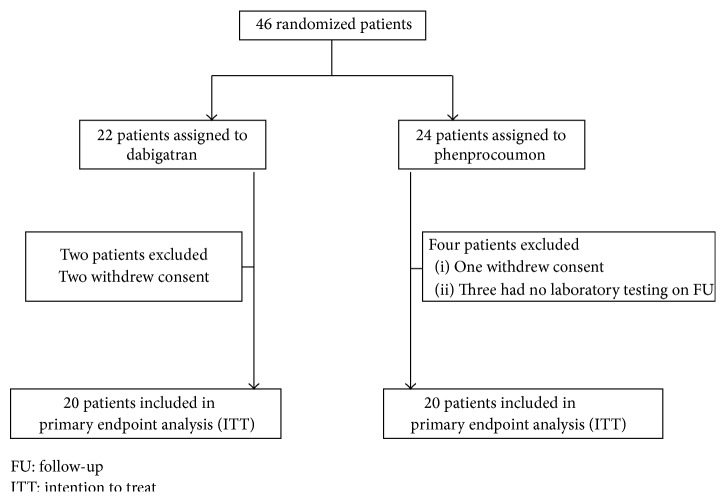
Patient population Dabi-ADP-2.

In Dabi-ADP-2, 46 patients concomitantly treated with clopidogrel were enrolled and randomized to receive dabigatran (*n* = 22) or phenprocoumon (*n* = 24). There was no significant difference in terms of baseline patient characteristics (Table 1). Median INR levels (interquartile range) at baseline were 1.2 (1.1-1.2) in the dabigatran group and 1.2 (1.1–1.4) in the phenprocoumon group. According to intention to treat analysis the primary and secondary endpoints could be analyzed in 20 patients in the dabigatran group and 20 patients in the phenprocoumon group (Figure 2).

### 3.2. Platelet Function Tests

**Figure 3:**
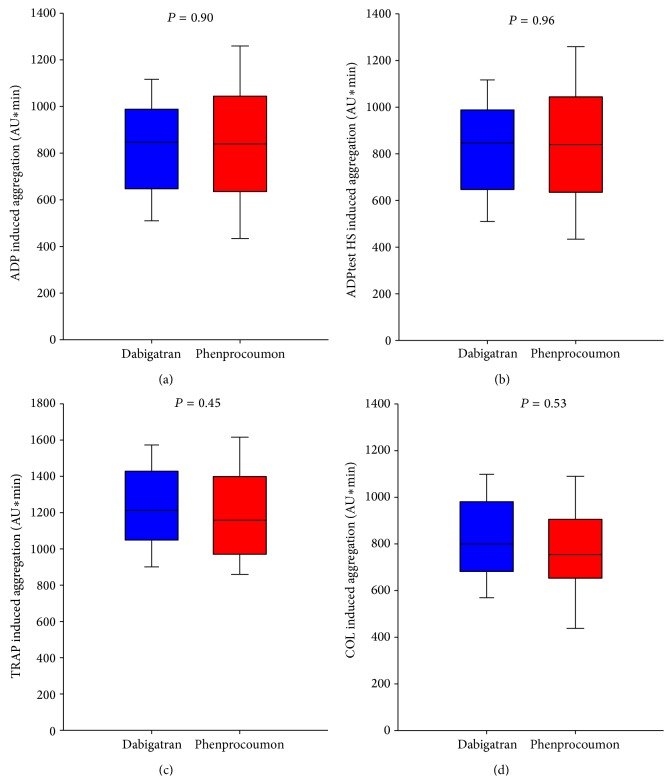
Platelet aggregation in patients with dabigatran and phenprocoumon therapy at 14 days (Dabi-ADP-1). Box plot analyses of multiple electrode platelet aggregometry (MEA) measurements for (a) ADP, (b) ADPtest HS, (c) TRAP, and (d) COL induced platelet aggregation in patients with either dabigatran (*n* = 30, blue) versus phenprocoumon (*n* = 32, red). Boxes indicate 25th and 75th percentiles and whiskers denote 10th and 90th percentiles.

**Figure 4:**
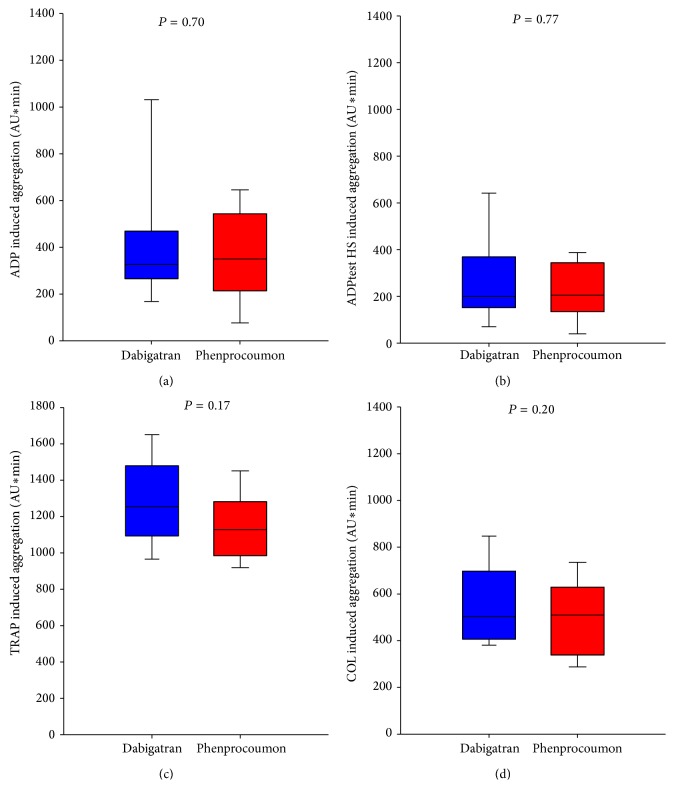
Platelet aggregation in patients with dabigatran and phenprocoumon therapy treated concomitantly with clopidogrel at 14 days (Dabi-ADP-2). Box plot analyses of multiple electrode platelet aggregometry (MEA) measurements for (a) ADP, (b) ADPtest HS, (c) TRAP, and (d) COL induced platelet aggregation in patients with either dabigatran (*n* = 20, blue) versus phenprocoumon (*n* = 20, red). Boxes indicate 25th and 75th percentiles and whiskers denote 10th and 90th percentiles.

There was no significant difference regarding the primary endpoint of ADP induced platelet function between patients treated with dabigatran as compared to patients with phenprocoumon treatment in either trial at 14 days (Dabi-ADP-1: 846 [650–983] AU × min versus 839 [666–1039] AU × min, *P* = 0.90 (Figure 3) and Dabi-ADP-2: 326 [268–462] AU × min versus 350 [214–535] AU × min, *P* = 0.70 (Figure 4)). There was also no significant difference regarding the secondary endpoints ADPtest HS, TRAP, and COL in either trial (Figures 3 and 4). Median INR values at follow-up were 1.2 (1.1–1.3) in the dabigatran group and 2.6 (1.9–3.3) in the phenprocoumon group in Dabi-ADP1 and 1.2 (1.1–1.4) in the dabigatran group and 3.0 (1.9–4.4) in the phenprocoumon group in Dabi-ADP-2.

In Dabi-APD-1 there was one patient in the dabigatran group who was switched to phenprocoumon and two patients in the phenprocoumon group who were switched to either rivaroxaban or enoxaparin by their primary physician during the study period (Figure 1). We therefore also analyzed patients according to the treatment they received. In the as treated analysis there was no significant difference regarding ADP induced platelet aggregation between patients treated with dabigatran (*n* = 29, as treated analysis) as compared to patients with phenprocoumon treatment (*n* = 31, as treated analysis) (850 [658–988] AU × min versus 842 [653–1044] AU × min, *P* = 0.94). There was also no significant difference regarding the secondary endpoints ADPtest HS (646 [529–760] AU × min versus 652 [457–855] AU × min, *P* = 0.96), TRAP (1195 [1049–1428] AU × min versus 1192 [1001–1399] AU × min, *P* = 0.57), and COL (804 [682–981] AU × min versus 752 [670–874] AU × min, *P* = 0.42).

### 3.3. Safety

In the phenprocoumon group of Dabi-ADP-2 there was one patient who suffered from stent thrombosis on day 13 due to clopidogrel discontinuation. In this patient ADP induced platelet aggregation was 1417 AU × min when he presented with STEMI to our emergency department. After clopidogrel loading and PCI of the culprit vessel he could be discharged and ADP values on follow-up under treatment with aspirin, clopidogrel, and phenprocoumon were 307 AU × min.

In both trials, no patient suffered from death, stroke, or TIMI minor or TIMI major bleeding during the follow-up period. The number of BARC Types 1 and 2 bleeding was low. In Dabi-ADP-1, there was one BARC Type 1 bleeding and 3 BARC Type 2 bleeding in the dabigatran group and none in the phenprocoumon group. In Dabi-ADP-2, one BARC Type 1 bleeding occurred in the dabigatran group and 2 in the phenprocoumon group.

## 4. Discussion

The main finding of our two randomized trials is that dabigatran as compared to phenprocoumon has no impact on ADP induced platelet aggregation in atrial fibrillation patients neither with nor without concomitant clopidogrel therapy. Furthermore there were no significant differences regarding the other mediators of platelet aggregation such as TRAP or collagen.

The hemostatic process is a dynamic, highly interwoven array of multiple processes [19] and antithrombotic agents inhibit specific steps in the coagulation cascade or in platelet aggregation. There have been some antithrombotic substances in the past, however, which have shown to induce a prothrombotic state through modification of pathways which differ from those who are primarily targeted, with the consequence of increasing adverse clinical events [20].

In our current Dabi-ADP-1 trial in patients with atrial fibrillation with no concomitant clopidogrel therapy, we could show that the direct thrombin inhibitor dabigatran has no impact on ADP, ADPtest HS, TRAP, and COL induced platelet aggregation as compared to phenprocoumon. As it is known, that phenprocoumon itself does not alter ADP induced platelet aggregation in patients solely treated with the vitamin K antagonist [21], our data suggests that dabigatran has no impact on ADP induced platelet function.

Our results are in line with the finding of former* in vitro* studies which could demonstrate no impact of dabigatran on ADP induced platelet aggregation in platelet rich plasma [3, 5]. Furthermore the values obtained for platelet function in our population treated with either dabigatran or phenprocoumon are similar to the normal reference intervals which were established in 117 healthy individuals [22].

Clinically, there is still an ongoing discussion regarding a prothrombotic effect of dabigatran. In the large multicenter RE-LY trial [1], concerns were raised that the rates of myocardial infarction were significantly increased in both tested dosages, which was debilitated after publication of a subsequent analysis of the RE-LY trial, where also silent MI were included [23, 24]. In contrast to this data, several meta-analysis of randomized trials with dabigatran came to the conclusion that dabigatran is associated with an increased risk of MI or ACS in a broad spectrum of patients [25–27]. The underlying pathological mechanism for this observation is still unknown.

Clopidogrel's efficacy may be hampered by genetic factors associated with clopidogrel metabolism as well as nongenetic factors such as patients' characteristics, comorbidities, and comedication [28]. One important example is the coumarin derivate phenprocoumon which is known to effectively reduce coagulation parameters but does not alter ADP induced platelet aggregation in patients solely treated with the vitamin K antagonist [21]. It is of interest however that concomitant treatment of phenprocoumon with DAT significantly attenuated the antiplatelet effects of clopidogrel in a previous study [10]. This is thought to be induced by a drug-drug interaction at the level of hepatic CYP metabolization, leading to a significant alteration of the vivo biotransformation of clopidogrel into its active thiol metabolite.

On the other hand, the parenteral direct thrombin inhibitor bivalirudin, as compared to unfractionated heparin, was able to further inhibit clopidogrel mediated ADP induced platelet aggregation [11, 12]. We therefore hypothesized that concomitant therapy with the oral direct thrombin inhibitor dabigatran might show a similar effect on ADP induced aggregation.

In fact, however, in our Dabi-ADP-2 trial in patients with atrial fibrillation with concomitant clopidogrel and aspirin therapy, we could show that the direct thrombin inhibitor dabigatran has no impact on ADP, ADPtest HS, TRAP, and COL induced platelet aggregation as compared to phenprocoumon.

These findings may be explained in several ways: Both phenprocoumon and dabigatran attenuate clopidogrel mediated ADP induced platelet aggregation in the same way or none of these substances have an impact on the platelet function even in the setting of clopidogrel therapy.

Data regarding the influence of dabigatran on clopidogrel mediated ADP induced platelet aggregation in patients is limited. Recently one small study with 12 healthy male individuals has evaluated concomitant therapy with dabigatran and clopidogrel on the pharmacokinetic and pharmacodynamic effect of either agent [6]. It was shown that neither ADP induced platelet aggregation nor the bioavailability of either agent was significantly altered by the combined therapy which is in line with our findings.

Our knowledge of the clinical role of new oral anticoagulants as part of triple therapy is still limited. So far one post hoc analysis of the RE-LY trial revealed that in the setting of concomitant single or dual antiplatelet therapy, dabigatran is associated with fewer bleeding events than warfarin [8] which are promising results. In our randomized study, duration of triple therapy with dabigatran was only 2 weeks and the number of patients was low. This does not allow for a meaningful assessment of the safety of this therapy. However, it is known that most of the bleeding events occur early after initiation of therapy [29]. We observed only few cases of Type 1 BARC bleeding.

*Study Limitations*. Limitations of Dabi-ADP-2 include the small sample size and the premature termination of the study. However results of our interim analysis implicated that in Dabi-ADP-2 more than 1000 patients needed to be enrolled and such a study is not feasible in such a patient population receiving triple antithrombotic treatment.

Secondly, baseline platelet function tests at randomization without any concomitant antithrombotic therapy were not available in all patients. Therefore our trial does not answer whether platelet function may have been affected between baseline and at two weeks when the primary endpoint was assessed.

Thirdly, aggregation was not tested with other agonists, such as thrombin-induced platelet aggregation, where a concentration-dependent inhibition with dabigatran has already been shown [3].

## 5. Conclusion

In conclusion, in these two randomized trials in patients with atrial fibrillation we could not find an impact on ADP induced platelet aggregation in patients treated with dabigatran as compared to phenprocoumon neither with nor without concomitant clopidogrel therapy.

*Clinical Perspective*. Traditional antithrombotic agents such as vitamin K antagonists as well as clopidogrel are currently challenged and will possibly be replaced by direct oral anticoagulants (apixaban, dabigatran, edoxaban, rivaroxaban) and newer P2Y12 inhibitors (prasugrel, ticagrelor). In the setting of triple therapy, choosing the right combination of antiplatelet and anticoagulation therapy therefore becomes more demanding as more options exist. Platelet function studies are useful to evaluate potential medication interactions which may attenuate their antithrombotic efficacy.

Clinically, limited data suggests that reductions in bleeding complications in this population may be achieved with the omission of aspirin [30], shorter therapy duration [31], or the use of dabigatran [8]. Newer more potent P2Y12 blocker are currently not recommended in the setting of triple therapy [32] as they may increase bleeding without reducing ischemic events [33]. Ongoing randomized studies such as PIONEER AF-PCI (ClinicalTrials.gov identifier: NCT01830543) or RE-DUAL PCI (ClinicalTrials.gov identifier: NCT02164864) evaluate also the role of newer agents and will further help to define the optimal treatment combination and duration in this challenging population.

## Figures and Tables

**Table 1:** Baseline characteristics of the Dabi-ADP-1 and Dabi-ADP-2 study population.

Characteristics	Dabi-ADP-1			Dabi-ADP-2		
	Dabigatran *n* = 35 (%)	Phenprocoumon *n* = 35 (%)	*P* value	Dabigatran *n* = 22 (%)	Phenprocoumon *n* = 24 (%)	*P* value
Age, years	69 ± 11	70 ± 13	0.89	71 ± 7	76 ± 8	0.06
Woman (%)	14 (40.0)	10 (28.6)	0.31	4 (18.2)	3 (12.5)	0.69
Arterial hypertension (%)	26 (74.3)	24 (68.6)	0.60	21 (95.5)	24 (100)	0.29
Diabetes mellitus (%)	3 (8.6)	9 (25.7)	0.11	5 (22.7)	9 (37.5)	0.28
Hypercholesterolemia (%)	23 (65.7)	23 (65.7)	>0.99	18 (81.8)	18 (75.0)	0.57
Prior myocardial infarction (%)	0 (0)	3 (8.6)	—	5 (22.7)	10 (41.7)	0.17
Prior bypass surgery (%)	1 (2.9)	1 (2.9)	>0.99	3 (13.6)	5 (20.8)	0.70
CHA_2_DS_2_-VASc score	2.8 ± 1.5	3.2 ± 1.6	0.25	4.0 ± 1.3	4.6 ± 1.0	0.13
Prior stroke (%)	0 (0)	1 (2.9)	—	1 (4.5)	1 (4.2)	1.0
Prior TIA (%)	0 (0)	1 (2.9)	—	0 (0)	1 (4.2)	—
Prior bleeding (%)	4 (11.4)	2 (5.7)	0.67	1 (4.5)	3 (12.5)	0.61
Medication at randomization						
Aspirin (%)	12 (34.3)	13 (37.1)	0.80	20 (90.9)	24 (100)	0.13
Clopidogrel (%)	0	0		22 (100)	24 (100)	—
Statin (%)	11 (31.4)	18 (51.4)	0.09	20 (90.9)	24 (100)	0.13
PPI (%)	4 (11.4)	7 (20.0)	0.51	5 (25.0)	8 (33.3)	0.55

## Abbreviations

AU: Aggregation units
DAT: Dual antiplatelet therapy
DOAC: Direct oral anticoagulants
HPR: High on-treatment platelet reactivity
MEA: Multiple electrode aggregation
MI: Myocardial infarction.

## References

[1] Connolly S. J., Ezekowitz M. D., Yusuf S. (2009). Dabigatran versus warfarin in patients with atrial fibrillation. *The New England Journal of Medicine*.

[2] Coughlin S. R. (2000). Thrombin signalling and protease-activated receptors. *Nature*.

[3] Wienen W., Stassen J.-M., Priepke H., Ries U. J., Hauel N. (2007). In-vitro profile and ex-vivo anticoagulant activity of the direct thrombin inhibitor dabigatran and its orally active prodrug, dabigatran etexilate. *Thrombosis and Haemostasis*.

[4] Eller T., Busse J., Dittrich M. (2014). Dabigatran, rivaroxaban, apixaban, argatroban and fondaparinux and their effects on coagulation POC and platelet function tests. *Clinical Chemistry and Laboratory Medicine*.

[5] Wong P. C., Jiang X. (2010). Apixaban, a direct factor Xa inhibitor, inhibits tissue-factor induced human platelet aggregation in vitro: comparison with direct inhibitors of factor VIIa, XIa and thrombin. *Thrombosis and Haemostasis*.

[6] Härtter S., Sennewald R., Schepers C., Baumann S., Fritsch H., Friedman J. (2013). Pharmacokinetic and pharmacodynamic effects of comedication of clopidogrel and dabigatran etexilate in healthy male volunteers. *European Journal of Clinical Pharmacology*.

[7] Schömig A., Sarafoff N., Seyfarth M. (2009). Triple antithrombotic management after stent implantation: when and how?. *Heart*.

[8] Dans A. L., Connolly S. J., Wallentin L. (2013). Concomitant use of antiplatelet therapy with dabigatran or warfarin in the Randomized Evaluation of Long-Term Anticoagulation Therapy (RE-LY) Trial. *Circulation*.

[9] Tantry U. S., Bonello L., Aradi D. (2013). Consensus and update on the definition of on-treatment platelet reactivity to adenosine diphosphate associated with ischemia and bleeding. *Journal of the American College of Cardiology*.

[10] Sibbing D., Von Beckerath N., Morath T. (2010). Oral anticoagulation with coumarin derivatives and antiplatelet effects of clopidogrel. *European Heart Journal*.

[11] Anand S. X., Kim M. C., Kamran M. (2007). Comparison of platelet function and morphology in patients undergoing percutaneous coronary intervention receiving bivalirudin versus unfractionated heparin versus clopidogrel pretreatment and bivalirudin. *The American Journal of Cardiology*.

[12] Sibbing D., Busch G., Braun S. (2008). Impact of bivalirudin or unfractionated heparin on platelet aggregation in patients pretreated with 600 mg clopidogrel undergoing elective percutaneous coronary intervention. *European Heart Journal*.

[13] European Heart Rhythm Association, European Association for Cardio-Thoracic Surgery, Camm A. J. (2010). Guidelines for the management of atrial fibrillation: the task force for the management of atrial fibrillation of the European society of cardiology (ESC). *European Heart Journal*.

[14] Wann L. S., Curtis A. B., Ellenbogen K. A. (2011). 2011 ACCF/AHA/HRS focused update on the management of patients with atrial fibrillation (update on dabigatran): a report of the American College of Cardiology Foundation Foundation/American Heart Association Task Force on Practice Guidelines. *Journal of the American College of Cardiology*.

[15] Sibbing D., Braun S., Jawansky S. (2008). Assessment of ADP-induced platelet aggregation with light transmission aggregometry and multiple electrode platelet aggregometry before and after clopidogrel treatment. *Thrombosis and Haemostasis*.

[16] Sibbing D., Stegherr J., Braun S. (2010). A double-blind, randomized study on prevention and existence of a rebound phenomenon of platelets after cessation of clopidogrel treatment. *Journal of the American College of Cardiology*.

[17] Anonymous http://www.timi.org/.

[18] Mehran R., Rao S. V., Bhatt D. L. (2011). Standardized bleeding definitions for cardiovascular clinical trials: a consensus report from the bleeding academic research consortium. *Circulation*.

[19] Furie B., Furie B. C. (2008). Mechanisms of thrombus formation. *The New England Journal of Medicine*.

[20] Topol E. J., Easton D., Harrington R. A. (2003). Randomized, double-blind, placebo-controlled, international trial of the oral IIb/IIIa antagonist lotrafiban in coronary and cerebrovascular disease. *Circulation*.

[21] Müller I., Massberg S., Zierhut W. (2002). Effects of aspirin and clopidogrel versus oral anticoagulation on platelet function and on coagulation in patients with nonvalvular atrial fibrillation (CLAFIB). *Pathophysiology of Haemostasis and Thrombosis*.

[22] Rubak P., Villadsen K., Hvas A.-M. (2012). Reference intervals for platelet aggregation assessed by multiple electrode platelet aggregometry. *Thrombosis Research*.

[23] Connolly S. J., Ezekowitz M. D., Yusuf S., Reilly P. A., Wallentin L. (2010). Newly identified events in the RE-LY trial. *The New England Journal of Medicine*.

[24] Hohnloser S. H., Oldgren J., Yang S. (2012). Myocardial ischemic events in patients with atrial fibrillation treated with dabigatran or warfarin in the RE-LY (Randomized Evaluation of Long-Term Anticoagulation Therapy) trial. *Circulation*.

[25] Uchino K., Hernandez A. V. (2012). Dabigatran association with higher risk of acute coronary events: Meta-analysis of noninferiority randomized controlled trials. *Archives of Internal Medicine*.

[26] Artang R., Rome E., Nielsen J. D., Vidaillet H. J. (2013). Meta-analysis of randomized controlled trials on risk of myocardial infarction from the use of oral direct thrombin inhibitors. *The American Journal of Cardiology*.

[27] Clemens A., Fraessdorf M., Friedman J. (2013). Cardiovascular outcomes during treatment with dabigatran: comprehensive analysis of individual subject data by treatment. *Vascular Health and Risk Management*.

[28] Sarafoff N., Byrne R. A., Sibbing D. (2012). Clinical use of clopidogrel. *Current Pharmaceutical Design*.

[29] Lamberts M., Olesen J. B., Ruwald M. H. (2012). Bleeding after initiation of multiple antithrombotic drugs, including triple therapy, in atrial fibrillation patients following myocardial infarction and coronary intervention: a nationwide cohort study. *Circulation*.

[30] Dewilde W. J. M., Oirbans T., Verheugt F. W. A. (2013). Use of clopidogrel with or without aspirin in patients taking oral anticoagulant therapy and undergoing percutaneous coronary intervention: an open-label, randomised, controlled trial. *The Lancet*.

[31] Fiedler K. A., Maeng M., Mehilli J. (2015). Duration of triple therapy in patients requiring oral anticoagulation after drug-eluting stent implantation: the ISAR-TRIPLE trial. *Journal of the American College of Cardiology*.

[32] Authors/Task Force Members, Windecker S., Kolh P. (2014). 2014 ESC/EACTS Guidelines on myocardial revascularization: The Task Force on Myocardial Revascularization of the European Society of Cardiology (ESC) and the European Association for Cardio-Thoracic Surgery (EACTS)Developed with the special contribution of the European Association of Percutaneous Cardiovascular Interventions (EAPCI). *European Heart Journal*.

[33] Sarafoff N., Martischnig A., Wealer J. (2013). Triple therapy with aspirin, prasugrel, and vitamin K antagonists in patients with drug-eluting stent implantation and an indication for oral anticoagulation. *Journal of the American College of Cardiology*.

